# Supplementary figures and images for: Species interactions in an Andean bird–flowering plant network: phenology is more important than abundance or morphology
Source: PeerJ. 2016 Dec 13;4:e2789. doi: 10.7717/peerj.2789 (PMC5157195; doi:10.7717/peerj.2789)

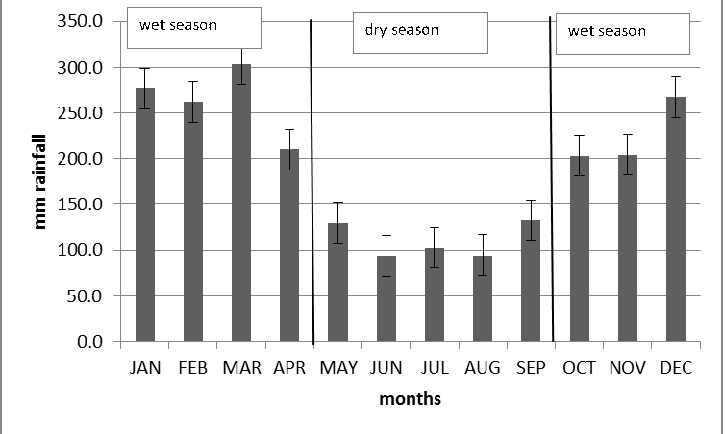

Supplement: Figure S1 [file peerj-04-2789-s001.png]

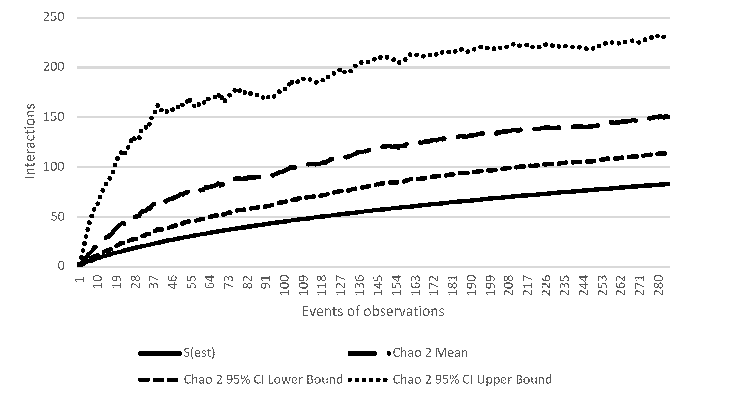

Supplement: Figure S2 — S(est) = Cumulative curve based on observed interactions. Chao2 = Mean Estimator Chao2; mean and confidence interval (95%) is shown. [file peerj-04-2789-s002.png]

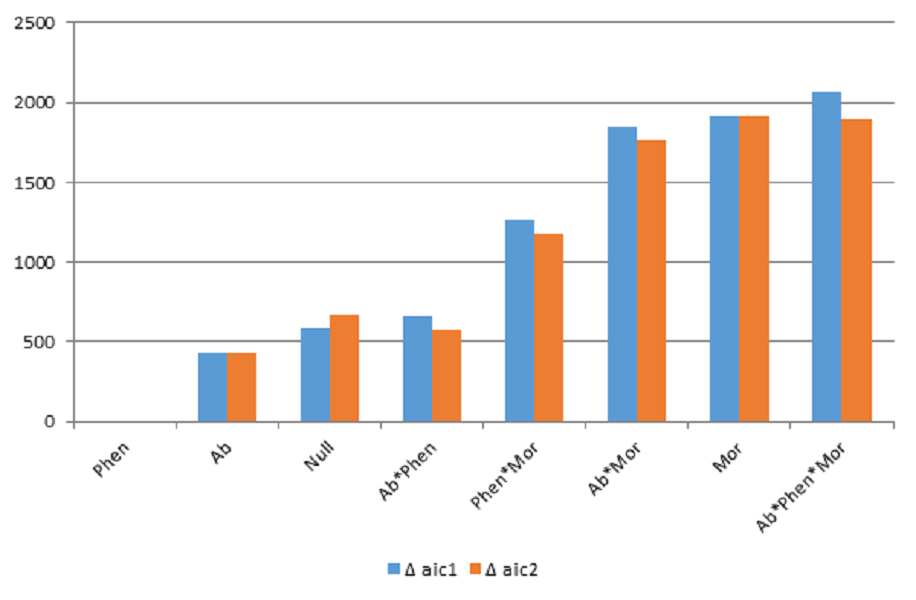

Supplement: Figure S3 — AIC1 was based on using number of species to determine number of parameters and AIC2 used number of matrices to determine number of parameters. In both cases, the phenology model had the lowest AIC value. Matrix codes: Ab, Abundance; Phen, Phenology; Mor, Morphology; Null, Null matrix. [file peerj-04-2789-s003.png]
