# Supplementary material for: Species interactions in an Andean bird–flowering plant network: phenology is more important than abundance or morphology
Source: PeerJ. 2016 Dec 13;4:e2789. doi: 10.7717/peerj.2789 (PMC5157195; doi:10.7717/peerj.2789)
Supplement: Table S1 [file peerj-04-2789-s004.docx]

### Table A1. R code for analysis

#Accompanying scripts have to be downloaded from the supplementary material of Vazquez, D. P., N. P. Chacoff, and L. Cagnolo. 2009. Evaluating multiple determinants of the structure of plant-animal mutualistic networks. Ecology 90:2039-2046

#Set working directory via File, Change dir"

library (bipartite)

#Create the Observed matrix (ObsMat) and the probability matrices (PhenMat, MorMat, AbMat)

ObsMat=read.table("TotObsMat.txt", header=TRUE)

PhenMat=read.table("TotPhenMat.txt", header=TRUE)

MorMat=read.table("TotMorMat.txt", header=TRUE)

AbMat=read.table("TotAbsMat.txt", header=TRUE)

#Create null model for Observation Interaction matrix ObsMat

I=27 #plants

J=17 #birds

red=ObsMat

red #Observation Interaction Matrix

red=as.matrix(red)

class(red)

pred=matrix(1/(I*J),I,J)

pred #Probability matrix or Null model, all interactions possible and equal

class (pred)

sum (red)

source("C:\\R\\Work\\R-Network_Oscar\\scripts\\quant2bin.r")

sum(quant2bin(red))

L=74 #distinct interactions

l=278 #total visits

#Run mgen script

source("C:\\R\\Work\\R-Network_Oscar\\scripts\\mgen.r")

ired=mgen(pred,l)

plotweb(ired)

class(ired)

sum(ired)

#Preparing for Netstat.r script (various versions)

source("C:\\R\\Work\\R-Network_Oscar\\scripts\\confint.r")

source("C:\\R\\Work\\R-Network_Oscar\\scripts\\intasymm.r")

source("C:\\R\\Work\\R-Network_Oscar\\scripts\\intereven.r")

source("C:\\R\\Work\\R-Network_Oscar\\scripts\\netstats.r")

#Netstats using Null Probablity Matrix as pmatr, pmatr=pred

netstats(rede,iter=1000,pmatr=pred)

AbMat2=as.matrix(AbMat)

PhenMat2=as.matrix(PhenMat)

MorMat2=as.matrix(MorMat)

#Netstats using Abundance Probablity Matrix as pmatr, pmatr=AbMat2

netstats(red,iter=1000,pmatr=AbMat2)

#Netstats using Phenology Probablity Matrix as pmatr, pmatr=PhenMat2

netstats(red,iter=1000,pmatr=PhenMat2)

#Netstats using Morphology Probablity Matrix as pmatr, pmatr=MorMat2

netstats(red,iter=1000,pmatr=MorMat2)

#Create Multiplicative Matrices Ab*Phen, Ab*Mor, Phen*Mor, Phen*Mor*Ab

AbPhen=AbMat2*PhenMat2

AbPhen

sum(AbPhen)

AbPhen1=AbPhen/sum(AbPhen)

sum(AbPhen1)

str(AbPhen1)

class(AbPhen1)

AbMor=AbMat2*MorMat2

sum(AbMor)

AbMor1=AbMor/sum(AbMor)

sum(AbMor1)

str(AbMor1)

class(AbMor1)

PhenMor = PhenMat2*MorMat2

sum(PhenMor)

PhenMor1=PhenMor/sum(PhenMor)

sum(PhenMor1)

str(PhenMor1)

class(PhenMor1)

AbPhenMor=AbMat2*PhenMat2*MorMat2

sum(AbPhenMor)

AbPhenMor1=AbPhenMor/sum(AbPhenMor)

sum(AbPhenMor1)

str(AbPhenMor1)

class(AbPhenMor1)

source("C:\\R\\Work\\R-Network_Oscar\\scripts\\netstats.r")

#Netstats with Abundance*Phenology probability matrix, AbPhen1

netstats(rede, iter=1000, pmatr=AbPhen1)

#Netstats with Abundance*Morphology probability matrix, AbMor1

netstats(rede, iter=1000, pmatr=AbMor1)

#Netstats with Phenology*Morphology probability matrix, PhenMor1

netstats(rede, iter=1000, pmatr=PhenMor1)

#Netstats with Abundance*Phenology*Morphology probability matrix, AbPhenMor1

netstats(rede, iter=1000, pmatr=AbPhenMor1)

#Create maximum liklihood functions, mlik

#dependent = rede, 1=prede, 2=AbMat2, 3=PhenMat2, 4=MorMat2, 5=AbPhen1, 6=AbMor1,

#7=PhenMor1, 8=AbPhenMor1

#number of parameters are 1, 44, 88, 132

source("C:\\R\\Work\\R-Network_Oscar\\scripts\\mlik.r")

aic1=mlik(rede,prede,par=1)

aic2=mlik(rede,AbMat2,par=44)

aic3=mlik(rede,PhenMat2,par=44)

aic4=mlik(rede,MorMat2,par=44)

aic5=mlik(rede,AbPhen1,par=44)

aic5=mlik(rede,AbPhen1,par=88)

aic6=mlik(rede,AbMor1,par=88)

aic7=mlik(rede,PhenMor1,par=88)

aic8=mlik(rede,AbPhenMor1,par=132)

#View results

aic1

aic2

aic3

aic4

aic5

aic6

aic7

aic8
